# Active inference with reusable state-dependent value profiles

**DOI:** 10.1007/s00422-026-01066-0

**Published:** 2026-10-09

**Authors:** Jacob Poschl

**Affiliations:** University of California, Santa Cruz, Santa Cruz, United States

**Keywords:** Active inference, Predictive processing, Free energy principle

## Abstract

Optimal behavior in volatile environments requires agents to deploy different value-control regimes across hidden latent structures. However, representing independent preferences, biases, and action confidence for every situation is computationally and statistically intractable. We introduce value profiles: a compact set of reusable parameter bundles—comprising outcome preferences, policy priors, and action precision—assigned to hidden states in a generative model. As posterior beliefs evolve trial-by-trial, effective control parameters emerge through belief-weighted mixing. This enables state-conditional strategy recruitment without the need for independent parameterization of every context. We evaluate this framework in a probabilistic reversal learning setting using simulation-based model recovery and parameter recovery, comparing profile-based models against static and uncertainty-coupled precision baselines. Model comparison favors the profile-based architecture, with consistent parameter recovery demonstrating its structural identifiability. Beyond predictive fit, we show how the framework allows for a mechanistic attribution of behavior to specific adaptive channels, distinguishing between changes in what an agent prefers and how decisively it acts. Overall, reusable value profiles provide a tractable computational account of belief-conditioned control, offering a mode-like representational scheme for behavioral flexibility that is both identifiable and theoretically grounded.

## Introduction

How should agents adapt *value updating* when environmental statistics change? In dynamic reward environments, optimal performance requires monitoring environmental statistics−especially volatility−to determine the *salience* of each new outcome and thus how strongly it should update action values (Behrens et al. 2007). A volatility-sensitive learner increases the effective learning rate after surprising outcomes (higher estimated volatility/uncertainty), enabling faster value revision in volatile regimes, whereas in stable regimes low volatility implies smaller trial-by-trial updates, preventing overreaction to stochastic outcomes (Behrens et al. 2007). Predictive processing and hierarchical Bayesian accounts formalize this trade-off as inference under a generative model, where *precision* (i.e., confidence-weighting of prediction errors) governs the gain on prediction errors and thereby shapes processes including learning and attention (Parr et al. 2022; Mathys et al. 2011; Schwartenbeck and Friston 2016; Feldman and Friston 2010). However, while many models posit that agents adjust control-relevant precisions (e.g., policy precision) in response to inferred context (e.g., changes in environmental statistics), the computational principles that determine *which control parameters should change, and how they should be assigned across latent contexts*, remain underspecified. Moreover, in many active inference implementations, key control parameters are classically treated as global or context-level quantities. While this can be powerful, such parameters are often empirically difficult to identify and to map onto mechanistic constraints.

This paper introduces value profiles: a state-conditional mechanism for reusable, identifiable motivated value control that avoids learning separate value settings for each inferred context. Rather than tuning value parameters globally or independently for each context, agents maintain a small library of profiles−bundled outcome preferences (*C*), policy priors (*E*), and (when needed) policy precision (γ)−that can be recruited jointly. Profiles are assigned to hidden states in the generative model, and as posterior beliefs over states evolve trial-by-trial, effective values emerge through belief-weighted mixing. This state-conditional recruitment supports adaptive behavior in changing environments while remaining structurally identifiable from behavior.

The profile framework is theoretically motivated by the fundamental constraint that agents cannot maintain independent preferences and precisions for each latent context they infer. Instead, they must compress the value space into a manageable library of reusable strategies / parameter bundles. Profiles provide this compression by bundling parameters into coherent behavioral strategies that can be shared across multiple hidden states. By reusing a finite set of profiles across many hidden states via belief-weighted recruitment, agents achieve computational efficiency while maintaining behavioral flexibility.

In tasks requiring rapid belief revision, such as probabilistic reversal learning, agents with profile-based value control should exhibit context-dependent behavioral strategies that emerge from belief-weighted profile recruitment. The specific adaptive mechanisms−whether through policy precision (γ), outcome preferences (C), or policy priors (E)−remain an empirical question. These adaptations should be belief-weighted and direction-specific−reflecting which state the agent infers (i.e., the identity of the inferred context), rather than merely how uncertain it is−thereby producing behavioral signatures that are difficult to capture with purely global or uncertainty-coupled adaptation.

Our contributions are threefold. First, we introduce value profiles as a parameter-sharing construction for state-conditional motivated value control: reusable bundles of control-relevant value parameters assigned to latent context states and recruited through belief-weighted mixing. Second, we show how profile recruitment induces smoothly varying effective outcome preferences and policy priors (and, when modeled as context-dependent, effective policy precision). Third, in a probabilistic reversal-learning task with latent context, we test whether this structure yields identifiable and predictively useful behavioral signatures; cross-validated model comparison favors profile-based control over static and uncertainty-coupled baselines.

The proceeding sections are as follows: section 2 reviews active inference and the control parameters relevant for value-conditioned action selection. Section 3 introduces reusable value profiles and the belief-weighted mixing scheme for deriving effective control parameters. Section 4 presents simulation-based model recovery and parameter recovery in a probabilistic reversal learning setting, comparing the profile architecture against static and uncertainty-coupled precision baselines. Section 5 reports model comparison and identifiability results, and Sect. 6 discusses implications, limitations, and future directions.

## Background

### Predictive processing and the free energy principle

Predictive processing formalizes perception, action, and learning as inference under a generative model (Friston 2009, 2010; Parr et al. 2022). Under the Free Energy Principle, agents minimize variational free energy through both perceptual inference and active sampling (Friston 2010). Adaptive behavior, however, depends not only on beliefs about hidden states but also on value structure—representations of which outcomes matter and how decisively to act. Precision weighting provides one influential account of such gain control, shaping which prediction errors drive learning and how decisively policies are selected (Schwartenbeck and Friston 2016). Yet the computational principles by which control-relevant quantities−especially precision weighting and prior preferences−are determined remain insufficiently characterized (Vance 2017; Torresan et al. 2025). In practice, specifying prior outcome preferences can be challenging, motivating approaches that learn preferences from experience (Sajid et al. 2021). We address this by proposing reusable *value profiles*: state-conditional bundles of outcome preferences, policy priors, and (when modeled as context-dependent) policy precision, assigned to hidden states and recruited through belief-weighted mixing as posterior beliefs over context evolve.

### Belief and value structures

Agents construct beliefs about hidden states through inference over sensory observations, updating their generative models as evidence accumulates (Parr et al. 2022). However, beliefs alone are insufficient for adaptive behavior: an agent must also represent which outcomes are desirable, which outcomes warrant attention, and how decisively to act (Friston et al. 2015). Values provide this evaluative structure, specifying preferred outcomes and the strength of those preferences within a given context. In this sense, inference and decision-making are organized around the attainment of preferred goal states, rather than being ends in themselves (Rigoli et al. 2017).

The environment presents a fundamental capacity problem: the space of possible states and contingencies far exceeds what any bounded agent could represent with independent parameters. If each situation required its own preference structure and precision setting, the resulting parameter space would be intractable for neural storage and for learning from finite experience. Because learning and planning with large/unknown state spaces already strain parameter capacity (Doshi-Velez 2009), we extend the same parsimony to value parameters by sharing them via reusable profiles rather than per-state tuning.

Reusable value profiles offer a compressive solution. Rather than independent parameterizations, agents maintain a small repertoire of coherent behavioral modes (e.g., exploration, exploitation, threat response) that can be deployed across multiple contexts. Each mode bundles together preferences (which outcomes to pursue), policy biases (which actions to favor), and precision (how decisively to act). In active inference, expected policy value and policy precision are tightly coupled—policy precision plays an inverse-temperature-like role and increases with expected value—so treating decisiveness as coordinated with evaluative control is well motivated (Friston et al. 2015). An exploratory mode, for instance, couples low decisiveness with preferences for informative outcomes and biases toward novel actions; an exploitative mode couples high decisiveness with outcome maximization and familiar action selection.

This bundling reflects the structure of adaptive behavior: attention, evaluation, and action selection must be coordinated to implement coherent strategies. By reusing a finite set of profiles across a larger state space, agents achieve computational efficiency while maintaining behavioral flexibility. This approach extends the factorization rationale of goal-directed Bayesian models, in which context, category, and action representations are distinct yet hierarchically related, and where model evidence trades off accuracy and complexity (Rigoli et al. 2017).

Precision in this paper is used in the broader predictive-processing sense of *gain control*—mechanisms that modulate the influence of prediction errors (Kanai et al. 2015; Friston 2010). In discrete active inference, one commonly isolated form is *policy precision* γ, which plays an inverse-temperature-like role in policy selection. For clarity, our profile framework treats outcome preferences (*C*), policy priors (*E*), and policy precision (γ) as distinct value-control components that can, in principle, vary across profiles.

### Active inference formalization

We implement this theoretical framework within discrete active inference, where agents maintain generative models over hidden states and observations (Smith et al. 2022). For our purposes, we distinguish between belief parameters that define the agent’s world model (likelihood mappings A, transition dynamics B, state priors D) and value parameters that determine preferences and action selection (outcome preferences C, policy priors E, and policy precision γ). While outcome preferences *C* can be equivalently expressed as priors within the generative model, distinguishing them in this way is useful for isolating which aspects of the model adapt across behavioral modes.

Value profiles bundle these preference and precision parameters (C, E, γ) into coherent units that can be assigned to hidden states, while the world model (A, B, D) remains shared across profiles. As beliefs about states fluctuate, effective values emerge through weighted mixtures of the assigned profiles. This provides a tractable way to implement state-conditional value control: rather than tuning preferences and precision independently for each state, agents reuse a small set of behavioral modes that share a common world model but differ in their evaluative and control parameters.

The specific assignment of active inference parameters to “belief” versus “value” categories is a modeling choice open to alternative interpretations. In hierarchical active inference architectures, for instance, value structure may be represented across multiple levels, with higher-level context states modulating lower-level preferences and precisions. Similarly, bundling *C*, *E*, and γ reflects our hypothesis about which value-control components co-vary during behavioral mode switching, though other groupings are possible. Here we adopt a single-level generative model (i.e., without explicit multi-level message passing) as a proof of concept, and show that state-conditional profile mixing yields identifiable, predictively useful behavioral signatures in volatile environments.

### Value profiles in relation to hierarchical active inference

Hierarchical active inference frames motivated control as solving multidimensional drive-to-goal problems, where action selection must be contextualized (e.g., by control context governing action–outcome contingencies and motivational context governing outcome desirability) and must resolve uncertainty via policy evaluation (Pezzulo et al. 2018). Here, we propose value profiles as an alternative architecture for the same class of problems: representing motivational context in a form that can systematically bias context-sensitive policy selection under uncertainty. In hierarchical active inference, higher-level (slower) context beliefs modulate lower-level preferences and precisions via explicit multi-level inference and message passing (Pezzulo et al. 2018). In contrast, profiles achieve context-sensitive modulation through belief-weighted recruitment of reusable parameter bundles. We validate this architecture’s empirical identifiability and theoretical coherence in controlled simulations (including probabilistic reversal learning), positioning profiles as a complementary approach that prioritizes parameter efficiency and mechanistic interpretability over compositional flexibility.

In hierarchical active inference, control is organized across levels of abstraction and timescale (Pezzulo et al. 2018). Higher-level latent beliefs encode slower-changing contextual states that constrain lower-level action selection by shaping the agent’s effective goals, while motivational context can modulate the precision (confidence) with which those goals bias policy selection (Pezzulo et al. 2018). This yields a principled account of motivated control: the same sensory evidence can lead to different action tendencies depending on inferred context and motivational state (Pezzulo et al. 2018; Rigoli et al. 2017).

Operationally, profiles compress hierarchical modulation into three architectural components: (i) a structured state space in which context factors modulate value assignments, (ii) an assignment matrix linking contexts to behavioral modes, and (iii) belief-weighted mixing that yields effective parameters as a function of posterior context beliefs. In this sense, profiles implement a lossy amortized form of hierarchical modulation: borrowing the idea of amortization from variational inference−where a learned mapping yields per-instance effective parameters instead of running iterative inference each time (Lin et al. 2018)−the agent maintains a compact repertoire of control regimes and recruits (or interpolates between) them as posterior context beliefs evolve trial-by-trial, rather than inferring control adjustments by explicitly reasoning over additional hierarchical layers (Pezzulo et al. 2018). This design trades the compositional flexibility of explicit hierarchical inference (Pezzulo et al. 2018) for structured parameter sharing that enhances identifiability and interpretability.

Although the formalisms differ, the conceptual correspondences are direct. Higher-level beliefs in hierarchical active inference that contextualize control correspond to the agent’s posterior beliefs over context states in the profile framework. The way higher levels constrain which outcomes and policies are favored corresponds to profiles encoding distinct patterns of priorities (e.g., information-seeking versus exploitation) via their value parameters. Hierarchical active inference emphasizes that motivation influences the strength and confidence of control; in profiles this is captured by regime-specific preferences and policy priors (and, when relevant, policy precision γ) that determine both what is pursued and how deterministically the agent commits to a policy. Where hierarchical active inference uses message passing between levels to propagate contextual influence (Pezzulo et al. 2018, 2015; Van De Maele et al. 2024), profiles use a structured mapping from context beliefs to profile recruitment, so that inferred context directly determines which control regime is expressed.

A productive way to interpret reusable profiles is as a constrained *repertoire* of cognitive agency: a small vocabulary of motivational–control regimes an agent can recruit. Hierarchical active inference proposes that cognition and control are organized by deep generative structure, where higher-order beliefs constitute an internal context (including motivational causes) that configures lower-level perception and action (Pezzulo et al. 2015). The profile perspective makes a complementary commitment: it treats control as composed of a small number of qualitatively distinct stances toward the world, and treats context inference primarily as determining which stance should govern behavior. On this view, hierarchical influence is not eliminated but *compressed* into reusable, interpretable regimes that are recruited through posterior beliefs over context. This compression aligns the model’s latent structure with psychological hypotheses about discrete behavioral modes (e.g., switching between information-seeking and exploitation), enabling mechanistic tests that are harder in more compositional architectures where control is distributed across many interacting degrees of freedom and is less identifiable from behavior.

Profiles and hierarchical active inference make different architectural tradeoffs. Profiles offer particular advantages when the scientific question is whether behavior can be explained by a *small repertoire of recurring regimes*—that is, a few qualitatively distinct control settings (e.g., information-seeking versus exploitation) that are reused across many trials and contexts. In such cases, strong parameter identifiability from limited behavioral data is often essential: by enforcing structured parameter sharing, profiles reduce the effective number of free degrees of freedom and thereby improve the recoverability of context-dependent adaptations from noisy choice sequences. The framework is especially well suited for tasks in which context structure is relatively simple (few distinct regimes) but must be inferred online from ambiguous observations, and where the goal is to test targeted hypotheses about *which* value-control components change across contexts (preferences *C*, policy priors *E*, and/or policy precision γ). By restricting the hypothesis space to reusable parameter bundles that are recruited through posterior context beliefs, profiles enhance both statistical identifiability and theoretical falsifiability: they make distinct mechanistic accounts correspond to distinct, testable patterns of behavior.

Conversely, explicit hierarchical (deep temporal) active inference is most useful when the domain exhibits nested structure across timescales—that is, when slower-changing contextual causes must provide higher-level constraints on faster dynamics, and when these constraints are implemented through explicit bidirectional message passing of predictions and prediction errors across levels (Kiebel et al. 2008; Parr and Friston 2018). Such temporal hierarchies can also support robust online inference by registering and “explaining away” surprising violations of expected temporal structure via supraordinate causes, thereby supplying contextual priors that stabilize inversion under uncertainty (Kiebel et al. 2008). Importantly, this is less a competitive comparison between frameworks than a difference in where hierarchical influence is implemented: reusable value profiles can be read as a lossy amortized form of hierarchical modulation, compressing contextual influence into a compact repertoire of parameter bundles that are expressed through belief-weighted recruitment (mixing) rather than by introducing additional levels and running explicit multi-level inference. On this view, the two approaches are continuous with one another—profiles emphasize structured parameter sharing to enhance empirical identifiability and mechanistic interpretability, whereas deeper hierarchies emphasize compositional flexibility across levels and timescales when the task demands it Parr and Friston (2018); Kiebel et al. (2008)—including the capacity for a supervisory level to intervene on lower-level control parameters in response to the agent’s own inferential state (Proietti et al. 2025).

The experiments in this paper test an architectural hypothesis, that context-dependent control can be explained by belief-weighted recruitment of a small library of reusable value profiles, and that this latent structure is identifiable from behavioral data even when context must be inferred from noisy observations. We therefore evaluate whether profile-based modulation is distinguishable from simpler alternatives that lack state-conditional structure, and whether recovered parameters preserve theoretically meaningful structure across independent fits.

### Related work and biological grounding

Neural control appears to rely on coordinated, context-sensitive modulation across multiple circuits rather than uniform global adjustment (Kanai et al. 2015; Ferguson and Cardin 2020). Dopamine has been proposed to encode precision (via postsynaptic gain) that effectively weights prediction errors in hierarchical active inference models; Friston et al. (2012). Separately, tonic dopamine has been modeled as reporting average reward/opportunity cost, thereby controlling response vigor and action latency (Niv et al. 2007). The LC–NE system has been argued to implement adaptive gain control that mediates an exploitation–exploration trade-off (Aston-Jones and Cohen 2005). Evidence suggests these systems operate in a context-sensitive manner: dopamine signals show phasic responses to unexpected outcomes that can promote behavioral adjustment, while response vigor in humans tracks recent average reward rate, with this coupling enhanced under L-DOPA, consistent with dopaminergic (putatively tonic) modulation of vigor (Beierholm et al. 2013). Higher-order thalamic nuclei, particularly the pulvinar, can gate cortical information flow (Sherman and Guillery 2003); in predictive-coding terms, this can be cast as modulating the gain (precision) of ascending prediction-error signalling (Feldman and Friston 2010; Kanai et al. 2015). In primates, single-unit recordings show that dopamine neurons initially respond to unexpected rewards, then transfer their phasic response to reward-predicting cues, and exhibit well-timed pauses when expected rewards are omitted—a pattern predicted by temporal-difference reward prediction error models (Schultz et al. 1997). Critically, these mechanisms are not well captured by a single *global* gain parameter: dopaminergic effects depend on which precisions are being modulated and where in a hierarchical architecture that modulation occurs (Friston et al. 2012), and dopaminergic responses depend on learned predictions, showing distinct patterns for better-/as-expected-/worse-than-expected outcomes (Schultz et al. 1997). This is in line with Bayesian accounts that link acetylcholine and norepinephrine to expected and unexpected uncertainty (Yu and Dayan 2005), and with hierarchical Bayesian learning models in which inferred volatility modulates precision-weighted, trial-wise updating (Mathys et al. 2011). Complementing neuromodulatory accounts, recordings in primate prefrontal cortex show context-dependent selection and integration of co-present inputs (e.g., motion vs. color) with little evidence for strong early gating of irrelevant inputs; analyses of population dynamics and recurrent-network modeling suggest this selection can be implemented by recurrent dynamics within the same prefrontal circuitry (Mante et al. 2013).

Active inference provides a formal framework for *adaptive control* through probabilistic inference, in which multiple parameter families can modulate effective learning and action. In particular, gain on sensory prediction errors shapes belief updating, while parameters governing policy selection shape how deterministically actions are chosen based on expected outcomes (Parr et al. 2022). Contemporary implementations have explored several approaches, including dynamic gain/precision adjustment based on inferred volatility or belief uncertainty (Mathys et al. 2014), schemes in which policy precision is itself optimized as a control signal that arbitrates between deliberative and habitual contributions to action selection (Proietti et al. 2025), and hierarchical schemes in which different levels operate at distinct timescales with bidirectional message passing (De Vries and Friston 2017; Friston et al. 2017). These models capture phenomena such as the explore–exploit trade-off and attentional modulation (Friston et al. 2015). However, many implementations either (i) treat key control parameters as global quantities shared across contexts or (ii) couple adaptation to generic uncertainty measures such as entropy over beliefs. While computationally tractable, these approaches face two limitations in complex environments with multiple distinct contexts: they can require many degrees of freedom when extended to richly structured tasks, and they do not naturally capture mode-like, coordinated shifts in strategy—such as threat response, exploratory foraging, or habitual control—observed in biological systems and human behavior.

Computationally, assigning independent gain or control parameters to each state/context can create identifiability problems: with limited behavioral data, a large number of weakly constrained parameters cannot be reliably estimated. Conceptually, purely global or uncertainty-coupled mechanisms are also insufficient to explain rapid switching between qualitatively distinct strategies when latent states change (e.g., shifting from cautious exploration to confident exploitation following a single informative observation). We hypothesize that biological systems may address this by recruiting reusable, coordinated control configurations rather than continuously retuning a single gain parameter.

## Methodology

### Generative model

We model behavior within a discrete partially observable Markov decision process (POMDP) framework. At each trial *t*, the agent observes outcomes $$o_t \in \{1,\ldots ,O\}$$, infers hidden states $$s_t \in \{1,\ldots ,S\}$$, and selects actions $$a_t \in \{1,\ldots ,A\}$$. The generative model is specified by three standard components: the likelihood matrix $$\textbf{A} \in [0,1]^{O \times S}$$ encoding p(o∣s), the transition matrix $$\textbf{B} \in [0,1]^{S \times S \times A}$$ encoding $p(s^{\prime }|s,a)$, and the prior $$\textbf{D} \in [0,1]^S$$ encoding $$p(s_1)$$.^1^ These belief parameters define the agent’s world model. In standard active inference, value parameters include outcome preference logits $$\textbf{C} \in \mathbb {R}^O$$ (pre-softmax log-prior parameters over outcomes), policy prior logits $$\textbf{E} \in \mathbb {R}^{|\Pi |}$$ (pre-softmax log-prior parameters over candidate policies $$\pi \in \Pi $$, i.e., action sequences), and policy precision $$\gamma \in \mathbb {R}_{>0}$$ (inverse temperature for action selection). In our implementation the planning horizon is two steps, so Π comprises all length-2 action sequences ($$|\Pi | = A^2$$). Policy-prior logits are specified at the level of individual actions as a vector $$\boldsymbol{\xi } \in \mathbb {R}^{A}$$ and lifted to the policy space by summing across timesteps, $$\textbf{E}_\pi = \sum _t \xi [\pi _t]$$. Because these logits are additively separable, the induced policy prior factorizes as a product of per-step action priors, $$\textrm{softmax}(\textbf{E})[\pi ] = \prod _t \textrm{softmax}(\boldsymbol{\xi })[\pi _t]$$, so the marginal prior over the immediately executed action is exactly $$\textrm{softmax}(\boldsymbol{\xi })$$.

### Value profiles

Each profile $k \in \{1,\ldots ,K\}$ bundles the standard active-inference value parameters into a single object:

1 $$\begin{aligned} \Omega _k \;=\; \{\textbf{C}_k,\textbf{E}_k,\gamma _k\}, \end{aligned}$$

with $\textbf{C}_k \in \mathbb {R}^O$, $\textbf{E}_k \in \mathbb {R}^{|\Pi |}$, and $\gamma _k \in \mathbb {R}_{>0}$. Outcome preferences determine which observations are desirable, policy priors encode habitual biases over action sequences, and precision controls how deterministically the agent selects actions. We bundle these parameters because they typically co-vary under coherent behavioral strategies.^2^ The logits $\textbf{C}_k$ and $\textbf{E}_k$ are mean-centered to fix the softmax gauge and improve identifiability, and are converted to probability distributions via softmax normalization when used during inference.

### State assignment and belief-weighted mixing

Profiles are assigned to hidden states through an assignment matrix $\textbf{Z} \in [0,1]^{S \times K}$, where each row sums to one: $\sum _{k=1}^K Z_{s,k} = 1$ for all *s*. Hard assignment sets each row to be one-hot (each state uses exactly one profile), while soft assignment allows convex mixing.

For multi-factor state spaces, profile assignment targets a specific state factor that represents behaviorally relevant context. In our implementation, profiles are assigned to the context factor (volatile vs. stable), while other factors (arm identity, action state) share profiles based on context beliefs. At each trial, the agent’s posterior beliefs over the assignment-relevant state factor $q_t(s_{\text {ctx}})$ determine which profiles are recruited. Profile weights are computed by pooling beliefs through the assignment matrix:

2 $$\begin{aligned} w_t(k) = \sum _{s_{\text {ctx}}=1}^{S_{\text {ctx}}} q_t(s_{\text {ctx}}) Z_{s_{\text {ctx}},k} \end{aligned}$$

These weights form a probability distribution over profiles: $w_t \in [0,1]^K$ with $\sum _k w_t(k) = 1$. Effective trial-wise parameters emerge through belief-weighted mixing:

3 $$\begin{aligned} &  \textbf{C}_t^{\text {eff}} = \sum _{k=1}^K w_t(k) \textbf{C}_k, \quad \textbf{E}_t^{\text {eff}} = \sum _{k=1}^K w_t(k) \textbf{E}_k,\nonumber \\ &  \gamma _t^{\text {eff}} = \sum _{k=1}^K w_t(k) \gamma _k \end{aligned}$$

**Fig. 1:**
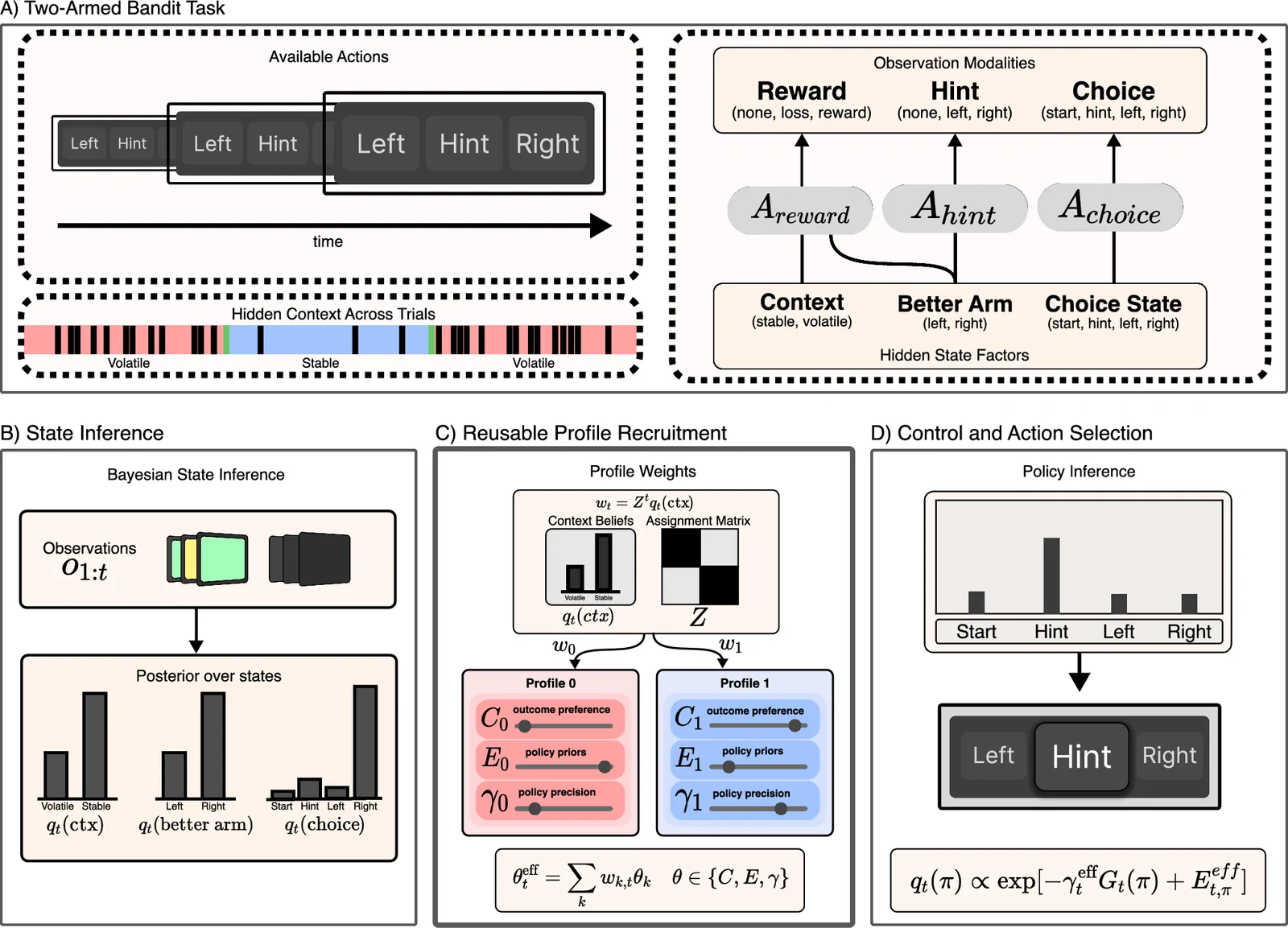
Task structure and the profile-based generative architecture. **(A)** Two-armed bandit task. On each trial the agent selects one of four actions (act_start, act_hint, act_left, act_right) and receives observations across three modalities: reward (none/loss/reward), hint (none/left/right), and choice confirmation (start/hint/left/right). Modality-specific likelihood mappings ($\textbf{A}_{\text {reward}}$, $\textbf{A}_{\text {hint}}$, $\textbf{A}_{\text {choice}}$) connect the three hidden state factors — context (stable/volatile), better arm (left/right), and choice state (start/hint/left/right) — to the observations they generate. The bottom strip depicts the latent context sequence across trials; context identity is never directly observed. **(B–D)** The inference and control pipeline in execution order: **(B)** Bayesian state inference yields posterior beliefs over all three factors; **(C)** the context posterior $q_t(s_{\text {ctx}})$ is mapped through the assignment matrix $\textbf{Z}$ to produce profile weights $w_t$, which mix the profile-specific value parameters ($C_k$, $E_k$, $\gamma _k$) into effective trial-wise parameters; **(D)** effective parameters enter the policy posterior, from which a policy is sampled and its first action executed

When beliefs strongly favor a single context state, the weighting concentrates on that state’s assigned profile. When context beliefs are uncertain, multiple profiles contribute proportionally, producing intermediate effective parameters. This mechanism makes value control state-conditional while maintaining continuous adaptation through belief-weighted mixing. We mix categorical natural parameters ($\textbf{C}_k$ and $\textbf{E}_k$ logits) linearly, yielding smooth interpolation between profile-defined action-value landscapes under uncertain context beliefs; for $\gamma _k$, linear mixing defines an effective inverse temperature under the same uncertainty. Fig. 1B–D illustrates the full pipeline: Bayesian state inference, belief-weighted profile recruitment, and the resulting policy inference.

### Inference and control

State inference is performed via standard Bayesian filtering for discrete (categorical) hidden states under the generative model defined above. Readers unfamiliar with discrete active inference may find the step-by-step treatment in Smith et al. (2022) a useful companion to this section. Given the previous posterior $q_{t-1}(s)$ and action $a_{t-1}$, a predictive prior is formed by propagating beliefs through the transition model:

$$ \tilde{q}_t(s) \;=\; \sum _{s'} B^{(a_{t-1})}_{s\,|\,s'}\, q_{t-1}(s'). $$

Upon observing $o_t$, beliefs are updated via Bayes’ rule,

$$ q_t(s) \;\propto \; A_{o_t\,|\,s}\,\tilde{q}_t(s), $$

followed by normalization. In the experiments reported here, the belief parameters $(\textbf{A},\textbf{B},\textbf{D})$ are treated as fixed and task-specified to isolate learning of value-profile parameters from behavior; in principle, they could also be learned (e.g., via standard active-inference/variational Bayes parameter learning), which we leave to future work. This posterior is then marginalized over the assignment-relevant factor and mapped through $\textbf{Z}$ to compute profile weights $w_t$, which yield effective parameters via mixing.

4 $$\begin{aligned} p(\pi \mid \text {history}_t) \propto \exp \left( -\gamma _t^{\text {eff}}\, G_t(\pi ) + E_{t,\pi }^{\text {eff}}\right) , \end{aligned}$$

where $G_t(\pi )$ denotes expected free energy.^3^

### Model specification, optimization, and identifiability

The full model is parameterized by $\Theta = \{\textbf{C}_k, \textbf{E}_k, \gamma _k, \textbf{Z}\}_{k=1}^K$, where each profile *k* has its own outcome preference logits, policy prior logits, and policy precision, and $\textbf{Z}$ specifies a state→profile assignment structure. In the current work, we adopt an estimation strategy optimized for model recovery and parameter identifiability.

For the generative phase, we instantiate this structured hypothesis class using task-informed anchored profile templates (e.g., volatile vs. stable regimes), which yields simulated behavior with known ground-truth parameters for recovery analyses. For the recovery phase, we employ exhaustive grid search over discretized parameter spaces (detailed in Sect. 4.3.2 and Table 2) rather than continuous optimization. This two-stage coarse-to-fine grid search systematically evaluates the constrained parameter space, is robust to local minima, and enables direct assessment of whether behavioral data can recover the assumed profile structure. While computationally expensive, this approach prioritizes reliability of parameter recovery over computational efficiency. In principle, profile parameters could also be estimated via MAP optimization of $\mathcal {L}(\Theta ) = \sum _t \log p(a_t \mid \Theta ) + \log p(\Theta )$ using constrained gradient-based methods; we leave this as future work.

To ensure identifiability, we impose structural constraints that remove degeneracies in the parameter space. Preference and policy prior logits are mean-centered before softmax transformation, eliminating translation invariance. Policy precisions are constrained to be positive ($\gamma _k > 0$) through the search space definition. Assignment matrix rows are constrained to the probability simplex ($\sum _k Z_{s,k} = 1$). These constraints remove common degeneracies (e.g., translation invariance of logits) and reduce non-identifiability by restricting parameters to a canonical gauge, thereby limiting transformations that can yield indistinguishable behavior. As in other mixture-like constructions, the model is invariant to permuting profile labels (label switching): relabeling *k* leaves the likelihood unchanged unless an ordering or anchoring constraint is imposed. In practice, we address this by evaluating recoverability of structural relationships (e.g., relative differences across profiles), and by fixing the state→profile mapping in M3 for the recovery experiments to remove label ambiguity.

## Experiments

### Task design and rationale

We evaluated the profile framework using a two-armed bandit task designed to create asymmetric context structure where different latent contexts require qualitatively different behavioral strategies. Figure 1A summarizes the task structure, including the available actions, observation modalities, and hidden state factors.

The environment consists of two volatility regimes that serve as hidden contexts that must be inferred from reward patterns. In the volatile context, the better arm yields reward with probability 0.70 and the worse arm yields reward with probability 0.30, with arm identities switching every 10 trials to create micro-reversals. This rapid switching necessitates frequent information-seeking through hint requests to track environmental changes. In the stable context, the better arm yields reward with probability 0.90 and the worse arm yields reward with probability 0.10, with arm identities remaining fixed within the context. The strong reward discrimination and temporal stability allow confident exploitation following minimal initial exploration.

On each trial, the agent selects from four actions: (act_left and act_right) generate binary reward outcomes according to the active context and current arm contingencies, act_hint produces a highly reliable cue about which arm is currently better (85% accuracy in both contexts), and act_start produces a null outcome. The agent receives three observations per trial: hint outcomes (which arm appears better), reward outcomes (win or loss), and choice confirmations (which action was taken). Critically, context identity is *not* directly observable−the agent must infer whether it occupies a volatile or stable regime from the pattern of reward probabilities it experiences. Agents maintaining beliefs over context states $(q_t(s_{\text {ctx}}))$ infer volatile contexts primarily from the moderate reward discrimination (70%/30%) characteristic of that regime, and stable contexts from the strong reward discrimination (90%/10%) characteristic of stability. While the environment features frequent arm reversals in the volatile context, the agent’s generative model accommodates these transitions through baseline uncertainty in the transition dynamics rather than relying on transition frequency as the primary cue for context inference. This separation between volatility regimes parallels Bayesian treatments that distinguish expected uncertainty within a regime from unexpected uncertainty arising from regime changes (Yu and Dayan 2005).

This design ensures that different contexts genuinely require different behavioral policies while also requiring inference to distinguish them. Critically, hint accuracy is identical across contexts (85%), eliminating confounds between context identity and information quality while maintaining uncertainty that requires active learning. The differential reward probabilities (70%/30% vs. 90%/10%) provide the statistical structure necessary for context inference through Bayesian belief updating. The asymmetric context structure provides an ideal testbed for evaluating whether agents can learn to infer hidden contexts and deploy context-specific behavioral strategies through belief-weighted profile mixing.

### Model specifications

We compared three model variants that differ in how they adapt control (value) parameters—in particular policy precision and, in the profile model, policy priors—while sharing identical generative structures ($\textbf{A},\textbf{B},\textbf{D}$) and task environments. To isolate the effects of value-parameter adaptation, all models used a shared, static transition model ($\textbf{B}$) with a fixed arm-switch probability (0.05) as a moderate compromise, rather than allowing context-dependent transition dynamics. This choice reflects the factorized transition parameterization adopted here, in which the task-relevant arm dynamics are represented independently of the latent context state, precluding direct dependence of better_arm transitions on context.^4^ All three models received the same three observation modalities (hints, rewards, choices) and inferred context from reward patterns. Table 1 summarizes the key differences in value-parameter control across models.

**Table 1:** Model specifications and precision control mechanisms

Model	Mechanism	Free Params	Uses Context	Key Limitation
M1	Static global	p=1	No	Fixed γ and preferences regardless of volatility; uncertainty is expressed only passively, through the entropy of the policy posterior induced by expected free energy, not through adaptation of any control parameter
M2	Entropy-coupled	p=2	No	Actively modulates only precision, not preferences; responds to local uncertainty (better-arm entropy) but not to context identity
M3	Profile mixing	p=6	Yes	Requires a structural prior over profile library and state → profile assignment Z (learning left for future work)

#### Model 1: static global precision

Model M1 maintains constant outcome preferences and policy precision across all trials, serving as a non-adaptive baseline. The agent uses fixed outcome preference logits $\boldsymbol{C_{\text {reward}}} = [0.0, -5.0, 5.0]$ for null, loss, and reward observations, and a fixed policy precision γ=2.5. During model recovery, only γ is treated as a free parameter (k=1), as outcome preferences are held constant to isolate precision effects. This model cannot adapt strategy based on context and uses the same exploration-exploitation balance regardless of environmental volatility. M1 performs Bayesian inference to update beliefs about which arm is currently better and whether the environment is volatile or stable, but these inferred context beliefs do not influence value parameters−precision and preferences remain fixed regardless of what the agent infers about environmental structure.

#### Model 2: entropy-coupled dynamic precision

Model M2 adapts policy precision dynamically according to belief entropy over the better-arm state factor:

5 $$\begin{aligned} \gamma _t = \frac{\gamma _{\text {base}}}{1 + \kappa \cdot H(\boldsymbol{q}_{\text {better\_arm}})}, \end{aligned}$$

where $H(\boldsymbol{q})$ denotes the Shannon entropy of beliefs over which arm is currently better. This produces exploratory behavior (low precision) when the agent is uncertain about arm identities, and exploitative behavior (high precision) when beliefs are confident. Outcome preferences remain fixed at $\boldsymbol{C_{\text {reward}}} = [0.0, -5.0, 5.0]$. Free parameters during fitting are $\gamma _{\text {base}}$ and κ (p=2). While M2 adapts based on belief uncertainty, it modulates only the precision parameter and responds to local uncertainty (which arm is better) rather than contextual structure (volatile vs. stable regime). M2 cannot learn that volatile and stable contexts require qualitatively different behavioral strategies such as different hint-seeking preferences. Although M2 infers context beliefs through standard Bayesian updating, it uses only better-arm uncertainty for precision modulation, not context identity itself.

#### Model 3: profile-based state-conditional mixing

Model M3 assumes a structural prior in which control parameters are organized into a small library of reusable value profiles (here K=2), each bundling policy precision $\gamma _k$ and policy-prior logits $\textbf{E}_k$. A state → profile assignment matrix $\textbf{Z}$ links profiles to the context factor (volatile vs. stable), and trial-wise effective parameters are obtained via belief-weighted mixing.

For data generation and interpretability in the recovery experiments, we anchor the two profiles to task-informed templates corresponding to exploratory/information-seeking versus exploitative control:

6 $$\begin{aligned} \text {Profile 0 (Volatile):} \quad&\gamma _0 = 2.0, \quad \textbf{E}_0 = [0.0, 3.0, 0.0, 0.0] \end{aligned}$$

7 $$\begin{aligned} \text {Profile 1 (Stable):} \quad&\gamma _1 = 4.0, \quad \textbf{E}_1 = [0.0, 0.5, 0.0, 0.0] \end{aligned}$$

where $\textbf{E}$ encodes policy-prior logits over $[\texttt {act} \texttt {\_start}, \texttt {act\_hint}, \texttt {act\_left}, \texttt {act\_right}]$.

During model fitting, free parameters include the two profile-specific precision values $\gamma _0$ and $\gamma _1$ as well as per-profile scaling factors for hint and arm preferences: $E_{\text {hint},0}$, $E_{\text {hint},1}$, $E_{\text {arm},0}$, and $E_{\text {arm},1}$ (p=6). Model M3 is the only variant that utilizes *inferred* context identity via $q_t(s_{ctx})$ to modulate both precision and policy preferences. As the agent accumulates evidence about reward probabilities, beliefs about context gradually shift. When context beliefs favor the volatile state (high $q_t(s_{ctx})[0]$), mixing weights shift toward Profile 0 (exploratory, information-seeking). When context beliefs favor the stable state (high $q_t(s_{ctx})[1]$), weights shift toward Profile 1 (exploitative, minimal hint-seeking). Critically, profile recruitment operates under inference uncertainty: early in a context period, when context beliefs are diffuse, profiles are mixed proportionally to belief strength rather than recruited deterministically. This supports context-dependent strategic adaptation that M1 and M2 cannot capture.

### Model recovery experiment

We evaluated the distinctiveness and identifiability of the profile-based formulation via a model recovery analysis. Specifically, we generated synthetic datasets from each candidate model and then fit all models to each dataset, testing whether the generating model is preferentially recovered under cross-validated predictive performance.

#### Data generation

We generated behavioral data from five sources: M1 with default parameters (γ=2.5), M2 with default parameters ($\gamma _{\text {base}} = 2.5$, κ=1.0), M3 with asymmetric profiles (Eq. 6–7) an epsilon-greedy baseline (ϵ=0.1, α=0.1), and a softmax Q-learning baseline (β=1.0, α=0.1) (Sutton and Barto 2018). The non-Bayesian baselines assess whether the active inference formulations can account for behavior generated under qualitatively different computational principles. If an active inference model were to explain non-Bayesian synthetic data comparably well, this would indicate excessive flexibility and weak structural constraints, undermining the interpretability of model comparisons. For each generator, we produced 5 independent runs of 400 trials with context reversals every 40 trials. Random seeds varied across runs to ensure independent samples.

#### Model fitting and cross-validation

For each generated run, we fitted all three active inference models using within-run 5-fold cross-validation. Trials were partitioned into five consecutive folds of 80 trials each. For each fold *f*, model parameters were selected by grid search on the remaining four folds and evaluated by held-out log-likelihood on fold *f*. We report the mean test log-likelihood and its standard error across folds. This design mitigates overfitting to idiosyncratic trial sequences, provides an empirical uncertainty estimate via fold-to-fold variability, and uses all trials efficiently by rotating them through training and test splits. In this setting, low fold variance is consistent with stable recovery, whereas elevated variance can indicate estimation instability or potential model misspecification.

Table 2 summarizes the parameter search spaces used for each model. All models employed a two-stage coarse-to-fine grid search to balance computational cost with parameter resolution.

**Table 2:** Parameter search spaces used for each model

Model	Parameters	Coarse Grid	Fine Grid
M1	γ	$\{0.5, 1.0, 1.5, 2.5, 4.0, 8.0, 12.0, 16.0\}$	7-point interpolation
M2	$\gamma _{\text {base}}, \kappa$	$\{0.5, 1.0, 1.5, 2.5, 4.0, 8.0\}\times \{0.05, 0.1, 0.25, 0.5, 1.0, 2.0, 4.0\}$	6×6 around best coarse point
M3	$\gamma _0, \gamma _1, E_{\text {hint},0}, E_{\text {hint},1}, E_{\text {arm},0}, E_{\text {arm},1}$	$\{1.0, 2.0, 2.5, 3.0, 4.0, 5.0\}^2 \times \{0.5, 1.0, 2.0, 4.0\}^2 \times \{0.5, 1.0, 2.0\}^2$	5,184 candidates (single stage)

#### Model selection criterion

We summarize model comparison using an information-criterion style score based on out-of-sample predictive performance. Specifically, we report

$$ \textrm{AIC}_{\textrm{CV}} \;=\; 2p \;-\; 2\,\textrm{LL}_{\textrm{test}}, $$

where *p* is the number of free parameters and $\textrm{LL}_{\textrm{test}}$ is the mean held-out (cross-validated) log-likelihood across folds.^5^ Lower $\textrm{AIC}_{\textrm{CV}}$ indicates a better trade-off between held-out predictive fit and parameter count.

#### Experimental hypotheses

We evaluated the proposed profile-based formulation using a controlled model-recovery design in which the data-generating model is known. This setting provides a direct test of (i) *structural discriminability* among candidate model classes under out-of-sample prediction, and (ii) *parameter identifiability* for the profile-based model.

**Hypothesis 1 (Selective model recovery):** Across generators, model comparison should be *selective*: each dataset should be best explained by the model class that generated it (lowest $\textrm{AIC}_{\textrm{CV}}$ / highest held-out log-likelihood). In particular, when data are generated by M3, M3 should outperform M1 and M2 despite its higher parameter count, indicating that the profile-based structure captures systematic choice regularities not reproduced by static or entropy-coupled precision alone. Conversely, when data are generated by M1 or M2, the corresponding simpler model should be preferred over M3, consistent with M3 not benefitting merely from increased flexibility but from its specific structural assumptions.

**Hypothesis 2 (Structural parameter recovery in M3):** When fitting M3 to M3-generated data, recovered parameters should preserve the *qualitative profile differentiation* implied by the generating templates. Concretely, recovered solutions should exhibit a stable between-profile separation in policy-prior strength (e.g., stronger hint-seeking bias in the volatile profile than in the stable profile), and where identifiable, a corresponding ordering in precision. We assess recovery primarily at the level of *structural relationships* (direction and relative magnitude of between-profile differences) rather than exact numerical equality, and we expect these relationships to be consistent across cross-validation folds and independent runs.

## Results

### Model recovery: asymmetric structure identification

Table 3 reports the $\textrm{AIC}_{\textrm{CV}}$ confusion matrix, where each row denotes the data-generating process and each column denotes the fitted candidate model; Fig. 3 plots the same values for the three active inference generators. Overall, recovery is asymmetric: M3-generated data are correctly identified (lowest $\textrm{AIC}_{\textrm{CV}}$ under M3), whereas data generated by M1 and M2 are preferentially captured by M1.

M3 is the only model that uses *inferred* context beliefs to modulate policy preferences via profile recruitment. When M3 generates data, agents shift between exploratory information-seeking as they infer volatile contexts under moderate reward discrimination (70%/30%), and exploitative direct action selection as they infer stable contexts under strong reward discrimination (90%/10%). In contrast, M1 uses fixed policy preferences regardless of inferred environmental structure, and M2 modulates only policy precision based on local uncertainty about which arm is better (i.e., entropy over the better-arm state), rather than beliefs about contextual volatility. These differences in how inferred contextual information enters control help explain why M1 and M2 yield substantially worse predictive fit on M3-generated action sequences.

M2 nevertheless improves over M1 on M3-generated data ($\textrm{AIC}_{\textrm{CV}}=173.04$ vs. 189.55), suggesting that entropy-coupled precision can capture some adaptive dynamics when uncertainty varies across trials. Concretely, M2 can become more exploratory when better-arm beliefs are uncertain and more exploitative when they are confident, which can partially align with the uncertainty patterns induced by volatility. However, because M2 cannot adapt policy preferences or condition control on inferred context identity, it does not reproduce the context-dependent strategy shifts expressed by belief-weighted profile recruitment in M3 ($\textrm{AIC}_{\textrm{CV}}=74.11$).

The asymmetry also appears in the opposite direction. When M1 generates the behavioral data, M1 achieves the lowest $\textrm{AIC}_{\textrm{CV}}$ (9.13) relative to M2 (17.44) and M3 (57.39). Similarly, for M2-generated data, M1 again attains the lowest $\textrm{AIC}_{\textrm{CV}}$ (12.10), outperforming both M2 (18.37) and M3 (56.78). The fact that M3 is strongly disfavored when the data-generating process does not require context-dependent strategy switching indicates that its success on M3-generated data is not attributable to generic flexibility. Instead, M3 provides a better description only when the underlying behavior contains the specific computational signature of belief-weighted profile recruitment; otherwise, its additional degrees of freedom are penalized and do not translate into improved predictive performance.

Notably, within the present task and estimation setup, M2 does not outperform M1 even on M2-generated data. This is plausibly attributable to the simplicity of the generative structure and the limited leverage available to entropy-coupled precision in this environment: when behavior is already well captured by fixed policy preferences with modest trial-to-trial variation, the additional precision-coupling parameter may not yield sufficient predictive gains to offset its complexity cost under $\textrm{AIC}_{\textrm{CV}}$. Importantly, this does not undermine the main recovery claim, that when context beliefs must be *inferred* and used to modulate policy preferences, M3 is uniquely supported by the data.

**Table 3:** $\textrm{AIC}_{\textrm{CV}}$ confusion matrix (mean ± SE across 5 runs). Lower values indicate better cross-validated fit. Bold indicates the lowest $\textrm{AIC}_{\textrm{CV}}$ within each generator row

Generator	M1	M2	M3
M1	**9.13 ± 0.34**	17.44 ± 0.40	57.39 ± 1.04
M2	**12.10 ± 0.78**	18.37 ± 0.57	56.78 ± 1.09
M3	189.55 ± 6.63	173.04 ± 8.08	**74.11 ± 2.01**
ϵ-greedy	231.53 ± 0.75	**228.45 ± 0.33**	249.22 ± 2.88
Softmax	**223.13 ± 0.39**	224.89 ± 0.41	240.78 ± 1.00

Table 4 reports the corresponding mean test log-likelihoods underlying the $\textrm{AIC}_{\textrm{CV}}$ comparisons. The log-likelihood results mirror the $\textrm{AIC}_{\textrm{CV}}$ patterns: on M3-generated data, M3 achieves substantially higher test log-likelihood (-31.05) than M1 (-93.78) or M2 (-84.52), indicating markedly better predictive accuracy for M3-generated action sequences. Conversely, on M1- and M2-generated data, M1 attains the highest test log-likelihood (-3.57 and -5.05, respectively), while M3 performs poorly (-22.69 and -22.39), confirming that M3 does not provide a uniformly better description across generators. Together, these results reinforce the interpretation that M3’s advantage is specific to data exhibiting context-dependent strategy modulation, rather than reflecting a generic capacity to fit diverse behaviors.

**Table 4:** Mean test log-likelihood (mean ± SE across 5 runs). Bold indicates the highest log-likelihood within each generator row

Generator	M1	M2	M3
M1	$\boldsymbol{-3.57 \pm 0.17}$	-6.72±0.20	-22.69±0.52
M2	$\boldsymbol{-5.05 \pm 0.39}$	-7.19±0.29	-22.39±0.55
M3	-93.78±3.31	-84.52±4.04	$\boldsymbol{-31.05 \pm 1.01}$
ϵ-greedy	-114.76±0.38	$\boldsymbol{-112.22 \pm 0.16}$	-118.61±1.44
Softmax	-110.56±0.19	$\boldsymbol{-110.44 \pm 0.20}$	-114.39±0.50

### Parameter recovery and structural preservation

To assess whether M3’s parameters are recoverable from behavioral data when context must be inferred, we examined parameter recovery when fitting M3 to M3-generated data.

Table 5 reports recovered parameters across the five cross-validation folds for a representative run. Throughout, Profile 0 corresponds to the *volatile* context and Profile 1 to the *stable* context (i.e., the canonical mapping used in the recovery experiments). Policy-prior logits are denoted by $\textbf{E}$ (over actions ordered as $[\texttt {start}, \texttt {hint}, \texttt {left}, \texttt {right}]$).

Across folds, the recovered solutions suggest that (for this task and hypothesis class) context-dependent changes in policy precision are not required to explain held-out behavior: the best-fitting configurations consistently assign similar γ to both profiles. In contrast, the fits preserve between-profile separation in policy priors for the volatile profile (Profile 0), which consistently recovers $\textbf{E}$ scaling [2.0, 0.5, 0.5] with elevated hint-seeking. This pattern is consistent with the asymmetric information value of the hint action in volatile versus stable regimes: volatile contexts consistently demand elevated information-seeking. In this setting, profile recruitment therefore appears to express adaptation primarily through policy-prior structure (via $\textbf{E}$) rather than through precision modulation, while leaving open that γ differences may become informative in tasks that more strongly load decisiveness or speed–accuracy tradeoffs.

Recovered parameters show high consistency across folds, with all folds selecting identical parameters across both profiles. Given that estimation was performed via exhaustive search over a discretized grid, this result should be interpreted as recoverability *within the examined parameterization*: the inferred behavior consistently supports a solution in which policy-prior differentiation across profiles is preserved, whereas asymmetric precision is not.

**Table 5:** Recovered M3 parameters across CV folds for a representative run. Generation templates: $\gamma _{\text {profile}}=[2.0,4.0]$ with Profile 0 = volatile and Profile 1 = stable, and $\textbf{E}_{\text {base}}=\{\textbf{E}_0,\textbf{E}_1\}$ where $\textbf{E}_0=[0,3.0,0,0]$ and $\textbf{E}_1=[0,0.5,0,0]$ over $[\texttt {start},\texttt {hint},\texttt {left},\texttt {right}]$ (hint-seeking prior logits). Reported $\textbf{E}$ scaling factors correspond to $[\texttt {hint},\texttt {left},\texttt {right}]$ for each profile

Fold	Recovered $\gamma _{\text {profile}}=[\gamma _0,\gamma _1]$	$\textbf{E}$ scaling (Profile 0)	$\textbf{E}$ scaling (Profile 1)
0	[5.0, 5.0]	[2.0, 0.5, 0.5]	[0.5, 0.5, 0.5]
1	[5.0, 5.0]	[2.0, 0.5, 0.5]	[0.5, 0.5, 0.5]
2	[5.0, 5.0]	[2.0, 0.5, 0.5]	[0.5, 0.5, 0.5]
3	[5.0, 5.0]	[2.0, 0.5, 0.5]	[0.5, 0.5, 0.5]
4	[5.0, 5.0]	[2.0, 0.5, 0.5]	[0.5, 0.5, 0.5]

Within this task and model class, these findings provide a mechanism attribution for profile-based value control: strategic shifts are best explained by changing which actions are preferred (hint-seeking versus direct arm selection) as inferred context beliefs change, rather than by changing how decisively actions are selected. More broadly, the profile framework admits multiple adaptive channels (e.g., policy priors and precision), and the present recovery results indicate that task structure can determine which channel is most strongly constrained by behavior.

### Mechanism attribution: profile dynamics in fitted models

**Fig. 2:**
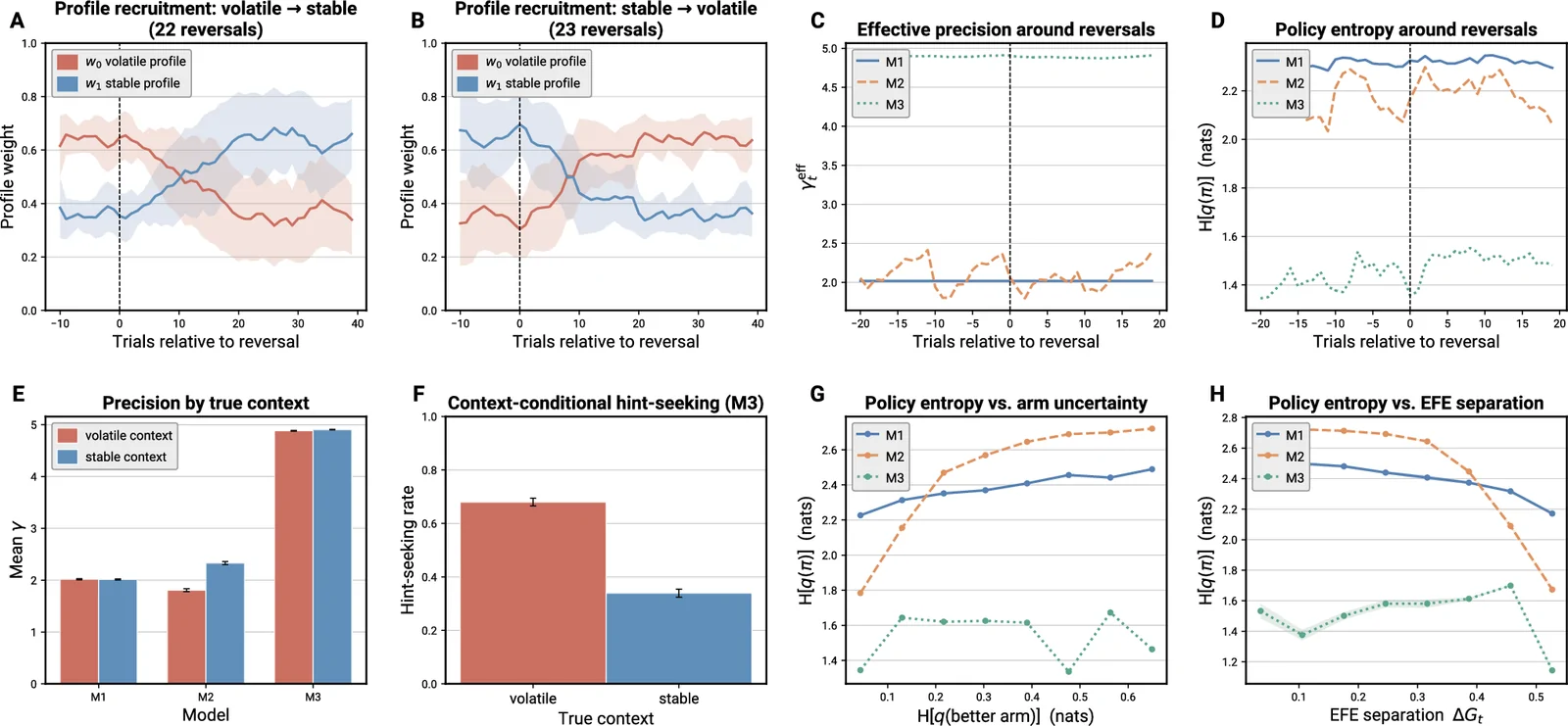
Mechanistic analyses of reusable profile recruitment under latent context inference. (A–B) Mean profile weights aligned to volatile-to-stable and stable-to-volatile context reversals. (C) Mean effective precision ($\gamma _t^{\text {eff}}$) aligned to context reversals. (D) Mean posterior policy entropy H[q(π)] aligned to context reversals. (E) Mean effective precision conditioned on true context. (F) Mean hint-seeking rate conditioned on true context (M3). (G) Mean posterior policy entropy as a function of uncertainty over the better_arm hidden state. (H) Mean posterior policy entropy as a function of the expected-free-energy separation between the best and second-best policy ($\Delta G_t$); shading is SEM

While model recovery establishes discriminability at the level of predictive fit, it does not by itself show which components of the fitted model instantiate the adaptation. We therefore analyze the internal dynamics of fitted M3 models to attribute adaptive behavior to profile recruitment and policy-preference mixing under latent context inference. Fig. 2 presents eight complementary analyses examining how recovered M3 parameters produce context-dependent behavioral strategies when context must be inferred from reward observations.

#### Profile recruitment at context boundaries

Panels A and B show profile-weight dynamics around context reversals when context must be inferred from reward outcomes. Following volatile-to-stable reversals (Panel A; trial 0), the weight on the volatile profile ($w_0$) decreases while the stable-profile weight ($w_1$) increases, with the opposite pattern after stable-to-volatile reversals (Panel B). Rather than switching instantaneously, profile weights transition over multiple trials as evidence accumulates for the new reward regime under latent-context uncertainty.

#### Precision dynamics and policy preference adaptation

Panel C tracks effective policy precision ($\gamma _t^{\text {eff}}$) in a 20-trial window around context reversals. In the fitted M3 model, policy precision remains stable across reversals, with only minor fluctuations during transitions. This reflects the recovered parameterization in which both profiles share the same policy precision. In contrast, M1 also exhibits approximately constant precision throughout, whereas M2 shows modest trial-to-trial modulation linked to uncertainty about which arm is currently better.

Panel D plots the corresponding posterior policy entropy H[q(π)] around the same reversals. Although M1’s precision is constant, its policy entropy is not: uncertainty reaches M1’s policy posterior passively, through the expected-free-energy landscape, because diffuse beliefs about the better arm compress the differences in $G_t(\pi )$ across candidate policies and thereby flatten q(π) without any control parameter changing. M2 shows the same passive dependence compounded by its active precision coupling, and M3’s entropy remains comparatively low and flat, reflecting its concentrated policy priors and higher recovered precision. Panels G and H characterize these relationships directly: M1’s policy entropy increases systematically with uncertainty over the better_arm state factor (Panel G) and decreases as the expected free energies of candidate policies separate (Panel H), despite fixed γ throughout; M2 exhibits steeper versions of both relationships, consistent with precision amplifying the passive effect. This distinction—between uncertainty expressed passively through the policy posterior and uncertainty actively recruiting changes in control parameters—is the sense in which Table 1 characterizes M1’s limitation.

Panel E summarizes the precision pattern by aggregating γ across all trials within each regime of the generating process, showing no systematic separation in precision between volatile and stable regimes for M1 or M3, and a modest separation for M2 inherited from its entropy coupling. Accordingly, the context-dependent behavioral differences expressed by M3 are better attributed to profile-specific policy preferences than to precision modulation.

Panel F isolates this mechanism in behavior: M3 exhibits substantially higher hint-seeking rates when the generating context is volatile than when it is stable. Note that trials in Panels E and F are binned by the true generating regime rather than by the agent’s context posterior; because context beliefs lag reversals by several trials (Panels A–B), this conditioning is conservative with respect to the belief-driven mechanism, as trials near reversals are assigned to the new regime while the agent’s recruited profile still reflects the old one. That the hint-seeking separation persists under this conservative binning indicates that belief-weighted mixing of profile-specific policy priors produces robust behavioral differentiation: inferred context beliefs recruit different preference profiles and thereby alter the relative propensity to select the hint action versus direct arm choices.

This pattern is consistent with the task structure: in volatile contexts, arm contingencies switch frequently, increasing the value of repeated information gathering, whereas in stable contexts the value of repeated hint requests diminishes once the better arm is identified. By design, M1 and M2 do not implement context-dependent shifts in policy preferences driven by inferred environmental structure: M1 holds policy priors fixed, and M2 modulates only precision. Together, this supports the interpretation that M3’s explanatory advantage in this setting derives primarily from preference adaptation via profile recruitment rather than from context-dependent precision changes.

### Non-Bayesian baselines

The ϵ-greedy and softmax Q-learning baselines produced behavior that none of the active inference models fit well ($\textrm{AIC}_{\textrm{CV}} > 223$ across active-inference fits for both baseline generators). This indicates that the active inference model family considered here is structurally constrained and does not flexibly match arbitrary action-generation rules outside its assumptions. Differences among active inference models on these baseline generators are small relative to the within-family recovery effects, as these baselines primarily serve as a scope check where the recovery experiment discriminates among active inference variants rather than to fit non-Bayesian agents.

**Fig. 3:**
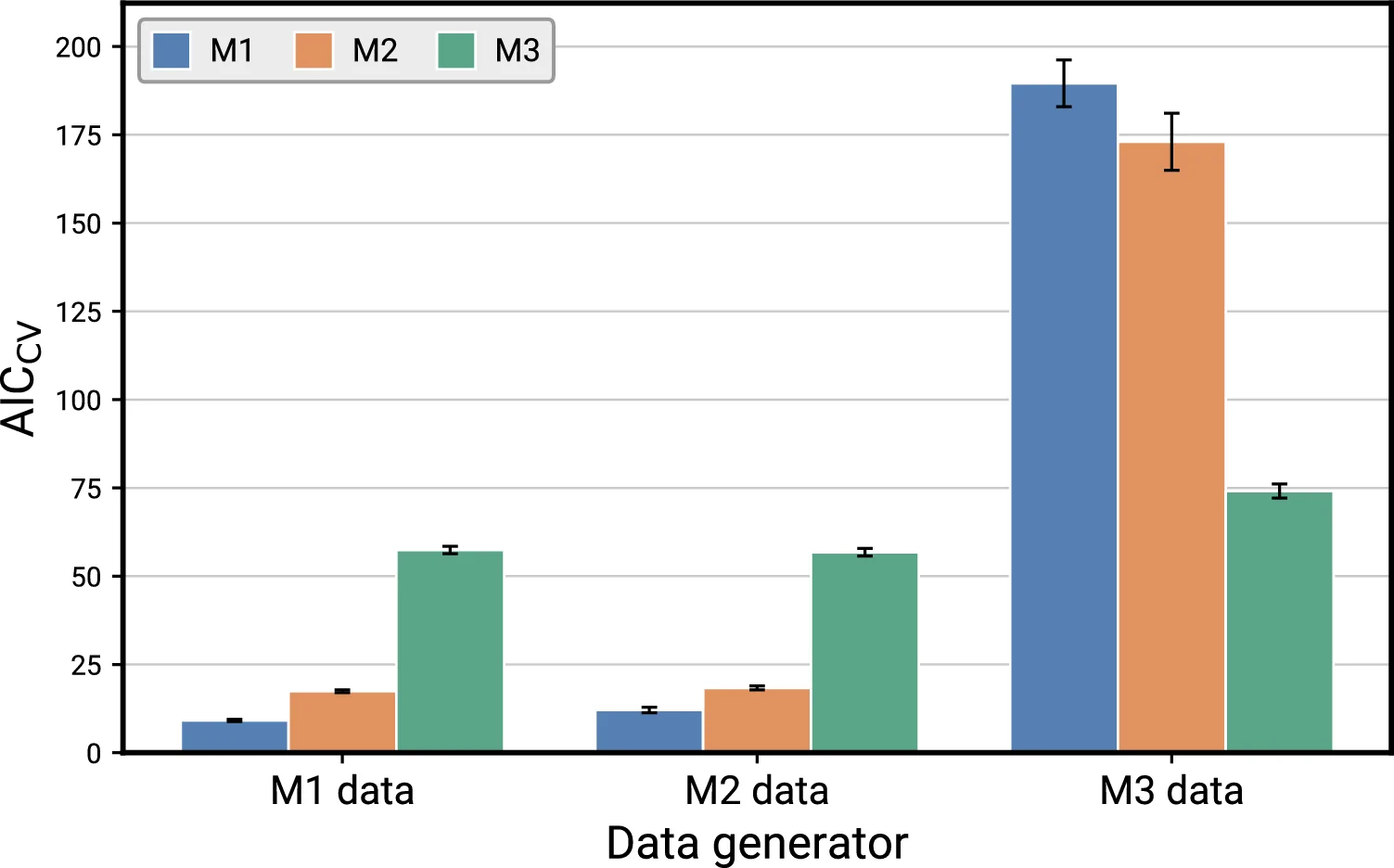
Model recovery results demonstrating asymmetric structure identification. $\textrm{AIC}_{\textrm{CV}}$ values (mean ± SE across 5 runs) for three active inference models fitted to data generated by each model

### Interpretation

The asymmetric recovery pattern supports three claims in this controlled recovery setting:

**Claim 1: Profile-based models express context-conditioned policy-preference adaptation.** When the generating process uses inferred context beliefs to recruit distinct behavioral strategies through policy-preference modulation, M3 provides the best predictive fit among the active inference candidates. In contrast, global precision (M1) or uncertainty-based precision modulation (M2) are less able to reproduce the resulting action sequences under the same observation and fitting assumptions. This pattern is consistent with profile recruitment implementing context-dependent changes in the relative desirability of information-seeking versus direct exploitation, rather than relying solely on static preferences or precision modulation tied to local uncertainty.

**Claim 2: Model complexity is appropriate, not excessive.** M3 is not uniformly favored across generators: when the data-generating process does not require context-dependent strategy switching, M3 is strongly disfavored relative to simpler alternatives. This indicates that M3’s additional parameters provide explanatory value primarily when the underlying behavior exhibits the specific computational signature of profile-based control, rather than reflecting generic flexibility or overfitting. Conversely, simpler models are appropriately preferred when the generating process is simpler, consistent with the intended role of information criteria.

The same pattern also highlights a task-conditional limitation of entropy-coupled precision adaptation: within this task and estimation setup, adding an entropy-coupling mechanism does not reliably improve predictive performance enough to justify its added complexity. This is not a central concern for the present claims, since the goal is to discriminate profile-based preference adaptation from alternative active inference mechanisms under a fixed task family; however, it does suggest that tasks offering greater leverage for decisiveness modulation may be required to robustly identify entropy-coupled precision effects.

**Claim 3: The framework is recoverable from behavioral data under inference uncertainty through policy-preference recovery.** Parameter recovery shows that the fitting procedure consistently identifies solutions that preserve the key structural property of the profile model—differentiated policy preferences across profiles—even when context must be inferred rather than observed. In the representative run reported above, cross-validation folds select the same grid-optimal configuration, indicating stable recovery within the examined discretized parameterization. Together with the mechanistic analyses, this supports the view that the profile framework makes behaviorally falsifiable predictions: when a task requires context-dependent strategies, model fitting can recover the latent preference structure that implements those strategies despite inference uncertainty.

Overall, these results place profile-based value control at an identifiable intermediate level of adaptive structure: more expressive than global or entropy-coupled precision schemes because it allows state-conditional preference modulation, yet more constrained than fully hierarchical formulations that introduce additional parameters across multiple timescales. Under latent context inference, profile recruitment provides a tractable mechanism for state-dependent adaptation through reusable behavioral modes, and the present recovery results indicate that its signature can be detected and attributed in held-out behavioral data.

## Discussion

### Policy precision versus policy prior adaptation: a task-specific outcome

Although the generative specification differentiated policy precision across profiles, the best-fitting recovered solutions in this task assigned the same policy precision to both profiles, with behavioral adaptation expressed primarily through profile-specific policy priors ($\textbf{E}$). Across recovery analyses (train/test partitions and random seeds), the grid-optimal solutions repeatedly selected equal γ across profiles, providing little evidence that asymmetric γ improves predictive fit in this environment.

Instead, adaptation is carried by differences in the recovered policy priors: the fitted profiles assign substantially different relative preference for hint-seeking versus direct arm selection. The mechanistic analyses (Fig. 2, Panel F) are consistent with this account, showing context-conditional hint-seeking behavior despite stable policy precision.

This outcome is naturally interpreted as task-dependent rather than as a general statement about the role of precision control. In the present environment, strategic control can be implemented effectively by shifting which actions are preferred (information seeking versus direct exploitation), which may reduce the need for decisiveness modulation via γ. By contrast, task families emphasizing response vigor, urgency, graded decisiveness, or lacking explicit information-seeking actions may provide stronger leverage for identifying precision-based adaptation, as in embodied choice settings where precision over the mapping to movement dynamics distinguishes cautious from impulsive strategies (Priorelli et al. 2025). A useful direction for future work is to vary these design factors systematically to characterize when profile recruitment is expressed primarily through policy priors versus policy precision.

### Limitations and future directions

Several methodological and theoretical limitations constrain the scope of our conclusions; each also suggests a concrete next step.

**Inference difficulty and contextual structure.** Although context must be inferred from reward outcomes, the inference problem we studied remains deliberately structured: there are two discrete regimes (volatile vs. stable), reward statistics are piecewise stationary within a regime, and regime transitions follow a simple schedule. The task is *hierarchical* in the limited sense that agents track both a higher-level regime and a lower-level “which arm is better” state. However, this is not the same as *multiple overlapping contextual factors* in the factorial sense (i.e., multiple latent causes simultaneously shaping outcomes and action values), nor does it address settings where regime boundaries are ambiguous, switching is stochastic, reward statistics drift gradually, or the number/identity of regimes must itself be learned.

A natural extension is to test profile recruitment under less structured regime dynamics (e.g., stochastic switching, gradual drift, or partially observable change points), and under genuinely factorial contexts (e.g., volatility and reward magnitude varying independently). Relatedly, while belief-weighted recruitment produces *continuous* mixing weights over discrete profiles, it does not constitute continuous *profile interpolation* in parameter space; future work could explore parameterized or manifold-based profile families that support smooth variation in the underlying control parameters themselves.

**Boundary conditions for control-channel recruitment.** In the present task family, recovered fits expressed adaptation primarily through changes in policy priors/preferences (e.g., shifting the relative desirability of information seeking versus direct exploitation) rather than through context-dependent changes in policy precision. This pattern is plausibly task-dependent, such that when adaptation can be implemented by changing *which* actions are preferred, decisiveness modulation may offer limited additional explanatory leverage. We did not systematically vary task features that are likely to differentially load these channels—such as reward discriminability, the informativeness and cost of information-seeking actions, the presence/absence of explicit information-seeking affordances, or demands for graded decisiveness (e.g., urgency or response-vigor tradeoffs).

A direct next step is a targeted task-family study that manipulates these design factors to map when adaptation is best captured by outcome preferences, policy priors/preferences, and decisiveness/precision control.

**Structure learning, estimation, and model comparison.** We impose a structural prior in which control parameters are organized into a small library of reusable profiles and linked to the latent regime factor through an assignment matrix $\boldsymbol{Z}$. For recovery experiments, we anchored the profile templates and fixed $\boldsymbol{Z}$ to the canonical mapping (volatile → Profile 0; stable → Profile 1) to isolate the contribution of belief-weighted recruitment and improve identifiability. In addition, parameter estimation used grid search over a discretized parameter set. This approach avoids dependence on gradient-based initialization, but it also limits scalability (parameter grids grow rapidly with additional parameters/profiles) and makes results contingent on the chosen grid resolution and priors.

The next logical direction is to relax these constraints by learning $\boldsymbol{Z}$ (and the number of profiles) from experience via variational or gradient-based methods with appropriate regularization, and comparing discretized search to continuous optimization under matched priors to assess robustness, identifiability, and computational tradeoffs as profile architectures become richer. Relatedly, our candidate set was restricted to models sharing an identical, fixed generative model, so that differences in held-out fit could be attributed to the value-modulation mechanism rather than to differences in world-model structure or learning dynamics. This design excludes two comparisons of independent interest: explicitly hierarchical formulations in which a supervisory level modulates lower-level control parameters, and standard active inference agents permitted to learn $\textbf{A}$, $\textbf{B}$, and $\textbf{D}$ from experience. Establishing when profile recruitment is preferable to either—in predictive accuracy, identifiability, or computational cost—requires a comparison in which the generative model is itself allowed to vary, and is a target for future work.

**Conflicting preferences across profiles.** The profiles recruited here differ in degree rather than direction: both favor reward, and differ in the relative desirability of information-seeking. A library containing *opposing* goal-directed bundles raises questions this task does not address. Because effective parameters are obtained by mixing natural parameters, diffuse context beliefs over conflicting outcome preferences yield an effective $\textbf{C}$ that flattens rather than preserving both goals; mixing normalized distributions instead would behave differently, and which rule is appropriate is a modeling question we leave open. Relatedly, although $\textbf{C}$ is bundled in $\Omega _k$, the present recovery experiments vary only $\textbf{E}$ and γ across profiles, so context-dependent outcome preferences remain formalized but untested here.

**Ecological validity and hypothesis testing in behavioral data.** We have not yet validated the framework on human or animal behavior. This is an important limitation, but also a clear opportunity, as the reusable-profiles formulation is particularly well suited for empirical hypothesis tests about cognitive modes since profiles provide *interpretable* candidate strategies and the inferred recruitment weights yield trial-by-trial predictions about when each mode is engaged. This makes it straightforward to test mechanistic questions such as whether volatility primarily shifts information seeking (preference changes), decisiveness (precision changes), or both.

Future work should fit the model to choice sequences from reversal-learning and volatility-manipulation paradigms, and then evaluate whether inferred profile recruitment predicts individual differences in flexibility or clinical dimensions, and whether neural/physiological correlates track mode engagement versus decisiveness modulation.

## Conclusion

A persistent challenge for predictive-processing and active-inference accounts is how agents can flexibly adjust value and control settings across latent contexts without maintaining a separate parameterization for every situation. We introduced *value profiles*, reusable bundles of control-relevant parameters assigned to latent states and recruited through belief-weighted mixing, as a tractable parameter-sharing construction for state-conditional adaptation. In controlled model-recovery experiments, the profile-based model was discriminable from global and entropy-coupled precision alternatives, providing superior penalized predictive fit when profile-based structure generated the data.

Within this task family, mechanistic analyses further indicated that the recovered adaptation was expressed primarily through profile-specific policy priors/preferences (i.e., context-conditioned action-type biases) rather than through context-dependent precision changes. This illustrates a central implication of the framework: profile recruitment can, in principle, support multiple adaptive channels, and the task structure determines which channel is most strongly constrained by behavior.

Conceptually, value profiles occupy an intermediate position between static global control and fully hierarchical active inference. Relative to mechanisms that express adaptation only through global or uncertainty-coupled modulation, profiles enable state-conditional shifts in strategy by recruiting distinct parameter bundles according to inferred context identity. Relative to richer hierarchical architectures that introduce separate parameters across multiple levels and timescales, the profile construction achieves context-sensitive control with a compact repertoire of reusable modes.

Overall, value profiles provide a compact and behaviorally testable route to state-conditional value control under latent context inference. Future work should extend the approach to settings with less structured regime dynamics, develop learning procedures that infer profile libraries and assignments from experience, and evaluate the framework against behavioral and neural data.

## Data Availability

The scripts for the data generated and analyzed in this study (simulation outputs and model-recovery) are available at: https://github.com/jacobposchl/active-inference-reusable-profiles.git

## Footnotes

1 For multi-factor state spaces and multi-modality observations, $\textbf{A}$, $\textbf{B}$, $\textbf{C}$, and $\textbf{D}$ generalize to object arrays with factor-specific or modality-specific components. We present single-factor notation for clarity; our implementation uses the full multi-factor formulation.

2 Intuitively, exploratory strategies often combine lower precision with preferences and priors that emphasize information gain/novelty, whereas exploitative strategies combine higher precision with preferences and priors favoring goal-directed, familiar actions (see Sec. 2.2).

3 We use the standard active-inference definition of expected free energy, in which $G_t(\pi )$ decomposes into (i) expected divergence between predicted outcomes and preferences (risk) and (ii) an information-gain term (epistemic value). Action selection samples a policy from this posterior and executes its first action (Parr et al. 2022).

4 In our reference implementation, this factorization corresponds to the standard factorized $\textbf{B}$ representation used in pymdp (Heins et al. 2022).

5 Classical AIC (Akaike 1974) is defined using the maximized in-sample log-likelihood; here we replace the in-sample term with a direct estimate of predictive performance from within-run cross-validation, yielding a single score that combines held-out fit with a standardized parameter-count penalty. We prefer an AIC-style criterion to BIC (Schwarz 1978) as our primary objective is predictive accuracy on held-out behavior rather than consistent identification of a true model under the assumption that it lies in the candidate set. Accordingly, BIC’s stronger plnn penalty is less well aligned with our goal in this setting.
